# Transcriptional diversity of the oxytocin receptor in prairie voles: mechanistic implications for behavioral neuroscience and maternal physiology

**DOI:** 10.3389/fgene.2023.1225197

**Published:** 2023-08-29

**Authors:** Joshua S. Danoff, Emma A. Page, Allison M. Perkeybile, William M. Kenkel, Jason R. Yee, Craig F. Ferris, C. Sue Carter, Jessica J. Connelly

**Affiliations:** ^1^ Department of Psychology, University of Virginia, Charlottesville, VA, United States; ^2^ Program in Fundamental Neuroscience, University of Virginia, Charlottesville, VA, United States; ^3^ Department of Psychological and Brain Sciences, University of Delaware, Newark, DE, United States; ^4^ Institute of Animal Welfare Science, University of Veterinary Medicine, Vienna, WIE, Austria; ^5^ Department of Psychology, Center for Translational Neuroimaging, Northeastern University, Boston, MA, United States

**Keywords:** oxytocin, alternative transcript isoforms, prairie vole (*Microtus ochrogaster*), DNA methyaltion, oxytocin receptor (OXTR)

## Abstract

The neurohormone oxytocin regulates many aspects of physiology primarily by binding to its receptor, the oxytocin receptor. The oxytocin receptor gene (*Oxtr*) has been shown to have alternative transcripts in the mouse brain which may each have different biological functions or be used in specific contexts. A popular animal model for studying oxytocin-dependent social behaviors is the prairie vole, a biparental and monogamous rodent. Alternative transcriptional capacity of *Oxtr* in prairie voles is unknown. We used 5′ rapid amplification of cDNA ends to identify alternative *Oxtr* transcription start sites in prairie vole brain tissue and uterine tissue. We then validated expression of specific transcripts in fetal brains and assessed the impact of exogenous oxytocin administration *in utero* on offspring brain development. We identified seven distinct *Oxtr* transcripts, all of which are present in both brain and uterine tissue. We then demonstrated that maternal oxytocin administration alters expression of a specific subset of *Oxtr* transcripts and that these different transcripts are under unique epigenetic regulation, such that in the perinatal period only one of the alternative transcripts is associated with DNA methylation in the *Oxtr* promoter. These data establish the existence of multiple *Oxtr* transcripts in prairie vole brain and uterine tissue and implicate oxytocin in the regulation of alternative transcript expression. These data have significant implications for our understanding of null mutant models in both mice and voles and translation in human birth and behavior.

## Introduction

Oxytocin is a neuropeptide hormone originally described for its role in promoting uterine contractions (Dale, 1906). Oxytocin signaling throughout the brain and body regulates many psychological and physiological processes including social behaviors, stress response, inflammation, and metabolism (Carter et al., 2020). Manipulation of oxytocin signaling early in life alters social behavior throughout the lifespan, including prosocial behaviors such as partner preference and parental behavior as well as aggression (Miller and Caldwell, 2015). Notably, oxytocin regulates many non-social behaviors including pain perception (Boll et al., 2018) and learning and memory (Chini et al., 2014). Physiologically, oxytocin regulates a wide array of processes, including bone development (Colaianni et al., 2014), cardiovascular development and regulation of blood pressure (Gutkowska and Jankowski, 2012), and regulation of metabolism and body weight, which is partly (but not completely) due to central effects of oxytocin in the neural control of food intake (McCormack et al., 2020). Accumulating evidence implicates aberrant oxytocin signaling in the pathophysiology of many neuropsychiatric disorders, including autism spectrum disorder, schizophrenia, mood disorders, and anxiety disorders (Cochran et al., 2013). In contemporary obstetric practice, oxytocin is used to induce and augment labor (Fox et al., 2015) and remains the first-line pharmaceutical for prevention and treatment of postpartum hemorrhage (World Health Organization, 2012). There is also interest in using oxytocin as a treatment for neuropsychiatric disorders including autism spectrum disorder, though these efforts have not been consistently successful (Huang et al., 2021; Sikich et al., 2021). Further examination of mechanism of action of oxytocin, including the genetic variability in its receptor, might improve pharmacological outcomes in behavioral disorders.

Oxytocin primarily acts by binding to its receptor, the oxytocin receptor (OXTR). In humans, OXTR is encoded by the *OXTR* gene, which is thought to consist of four exons and three introns (Inoue et al., 1994). To some extent, homology has been used in model organisms to define transcript structure, however, a recent study of epigenetic regulation of *Oxtr* in mouse hippocampus identified eight isoforms of *Oxtr* which originate from alternative transcription start sites, alternative splicing of exons, or both (Towers et al., 2018). Two of the isoforms have the potential to be translated into full length OXTR proteins while the remaining six would make partial proteins, if translated. An important model system for examining oxytocin-dependent social behaviors are prairie voles, which display a wide range of human-like social behaviors including pair bonding and biparental care and have a highly conserved *Oxtr* gene (Tabbaa et al., 2017; Perkeybile et al., 2019). In previous studies, we have shown that DNA methylation in *Oxtr* is predictive of gene expression in the brain (Danoff et al., 2021). However, the assay for gene expression in this study measured only two exon splicing events of *Oxtr*, each of which could represent one or more transcripts. We have previously identified the presence of one alternative transcript, *Oxtr*-H, in the prairie vole nucleus accumbens (Danoff et al., 2021). Given the findings of multiple *Oxtr* transcripts in the mouse brain, we sought to establish the presence of additional alternative transcripts in prairie voles.

In this study, we use 5′ rapid amplification of cDNA ends (5′ RACE) to identify *Oxtr* transcripts present in prairie vole nucleus accumbens and uterus. We identify seven alternative *Oxtr* transcripts containing the annotated fourth exon, each of which have a different transcription start site. We show that exogenous oxytocin exposure at birth differentially impacts *Oxtr* variant expression in the fetal brain, suggesting distinct biological functions of each of the transcripts. Finally, we provide evidence that DNA methylation in an important *Oxtr* regulatory region regulates only specific transcripts.

## Methods

### Animal model

All subjects were laboratory-bred prairie voles (*Microtus ochrogaster*) derived from wild-caught stock near Champaign, Illinois treated as previously described (Perkeybile et al., 2019). Subjects are at least four generations removed from wild-caught stock. Breeding pairs were housed in polycarbonate cages (44 cm × 22 cm × 16 cm). Same sex sibling pairs were housed in polycarbonate cages (27 cm × 16 cm × 16 cm) after weaning on postnatal day 20 (PND20). Animals were given food (high-fiber Purina rabbit chow) and water *ad libitum*, cotton nestlets for nesting material, and were maintained on a 14:10 light:dark cycle.

### Tissue for 5′ rapid amplification of cDNA ends

Four nucleus accumbens RNA samples (two male and two female, aged between postnatal days 21 and 24) from our previous study of early life experience were pooled for 5′ RACE (Perkeybile et al., 2019). Uterine samples were taken from two adults: one virgin (115 days old) and one postpartum day 1 (multiparous and over a year old) from our colony at the University of Virginia. Uterine samples were collected from animals that were deeply anaesthetized with an overdose of Euthasol (Covetrus) and sacrificed via cervical dislocation. An incision was made on the abdomen and the uterus was removed and flash frozen on dry ice, then stored at −80°C until nucleic acid isolation. RNA was isolated using an AllPrep DNA/RNA Mini kit (Qiagen) according to manufacturer instructions. All procedures for collection of brain tissue were approved by the IACUC at the University of California, Davis. All procedures for collection of uterine tissue were approved by the IACUC at the University of Virginia.

### 5′ rapid amplification of cDNA ends

**FIGURE 1:**
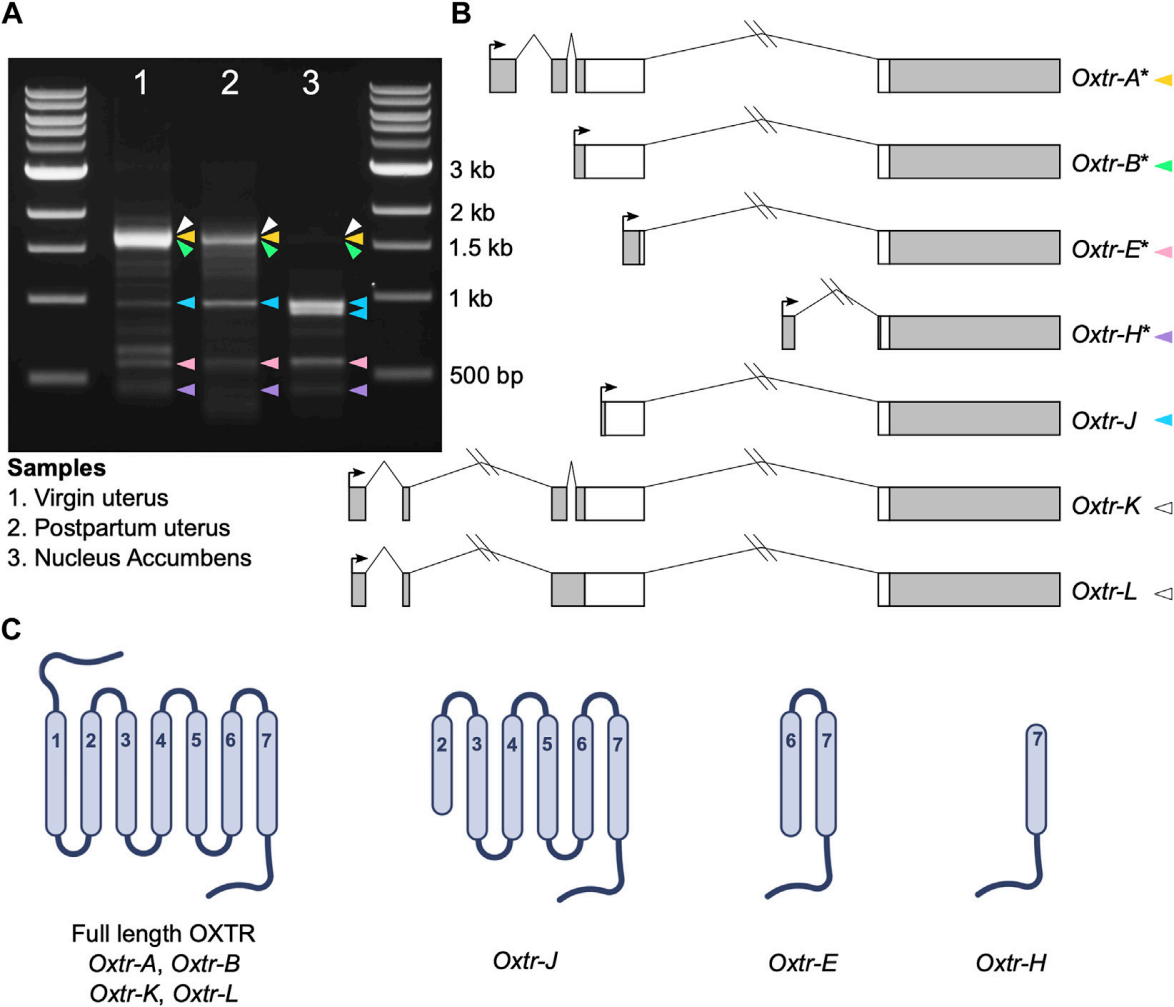
*Oxtr* transcripts identified in prairie vole uterus and nucleus accumbens. **(A)** Gel electrophoresis image showing the bands produced by 5′ RACE in adult virgin uterus (lane 1), postpartum uterus (lane 2) and juvenile nucleus accumbens (lane 3). Colored arrowheads indicate bands with specific *Oxtr* RACE products and match colors in Figure 1B. **(B)** Schematics of *Oxtr* transcripts identified by 5′ RACE. Exons are indicated by boxes (coding, white; untranslated, gray). Introns are shown with solid lines. Transcription start sites are indicated by arrows. Transcripts previously identified in mouse hippocampus are indicated by an asterisk. **(C)** Schematics showing the potential protein structures of each of the transcripts.

5′ RACE was completed using the GeneRacer Kit with Superscript III RT (Invitrogen) according to manufacturer instructions. For uterine samples, 3 μg of RNA was used as input. For nucleus accumbens, 2.5 μg, which was pooled from four different samples, was used as input. Briefly, RNA was dephosphorylated and decapped and the GeneRacer oligo was ligated to the mRNA. mRNA was transcribed into cDNA using Superscript III RT using random primers. Touchdown PCR was performed with Platinum *Taq* DNA Polymerase High Fidelity (Invitrogen) according to manufacturer instructions with an annealing temperature of 65°C, with the provided forward GeneRacer primer, and the reverse gene-specific primer 5′-CGT​CTC​TCC​GGG​CCT​GCT​GCC​CT-3′. A nested PCR was then performed using 1 μL of initial PCR as input, the provided nested forward GeneRacer primer, and the nested reverse gene-specific primer 5′- CCG​GGC​CTG​CTG​CCC​TTC​AGG​T-3′ according to manufacturer instructions. 20 μL of nested PCR product was run on a 1.5% agarose gel with ethidium bromide. Single bands denoted in Figure 1 were cut from the gel and DNA was extracted using the QIAquick Gel Extraction Kit (Qiagen) and resuspended in 10 μL of Buffer EB. Nonspecific PCR bands were identified by: 1) PCR controls missing either template, forward primer, or reverse primer; 2) an experiment where we denatured PCR products and slowly cooled them to prevent PCR product hybrids; and 3) sequencing. We performed PCR and downstream cloning and sequencing using 5′ RACE products on three separate occasions and present sequencing data from all three runs. However, data are compiled into one figure for visualization purposes.

### TOPO TA cloning, plasmid minipreps, and sequencing

Each gel-extracted band represents a PCR product that was cloned using the TOPO TA Cloning Kit (Invitrogen) according to manufacturer instructions. Briefly, 4 μL of the purified PCR product was cloned into the TOPO vector. One Shot TOP10 *E. coli* was transformed using the heat shock method. Transformed *E. coli* were spread on LB-agar plates containing 50 μg/mL ampicillin for selection and incubated at 37°C for 24 h. Three single colonies from each PCR product were picked and grown overnight in liquid LB with 50 μg/mL ampicillin at 37°C shaking at 200 rpm. Cells were pelleted by spinning at 4000 rcf for 10 min and plasmids containing 5′ end clones were isolated using the PureLink Quick Plasmid Miniprep Kit (Invitrogen). Plasmids were sent to Genewiz for sequencing. Sequences were aligned to *Oxtr* using NCBI’s blastn. Sequence information for each transcript is provided in Supplementary Data.

### Tissue from induction of labor study

Methods for generation of samples for the induction of labor study and measurement of DNA methylation in these samples were previously reported and are summarized below (Kenkel et al., 2019). Prairie voles were paired and allowed to mate using a timed-mating paradigm which enabled prediction of the day of birth. On the expected day of birth, pregnant dams were intraperitoneally injected with either saline (*n* = 9 dams), low dose oxytocin (0.125 mg/kg, *n* = 6 dams), medium dose oxytocin (0.25 mg/kg, *n* = 8 dams), or high dose oxytocin (0.5 mg/kg, *n* = 6 dams). An additional group of pregnant dams were not injected with anything and used as a no treatment control (*n* = 9 dams). 90 min following injection, pregnant dams were anesthetized with isoflurane and fetuses were removed and euthanized. Fetal brains were extracted and sectioned into thirds (which roughly correspond to forebrain, midbrain, and hindbrain), then they were flash frozen on dry ice. DNA and RNA were extracted from the midbrain of one male and one female fetus per mother using AllPrep mini kits (Qiagen). All procedures for the induction labor study were approved by the IACUC at Northeastern University.

### Gene expression analysis in induction of labor study samples

Offspring used for gene expression analysis were restricted to animals with G/G genotype at SNP KLW2. This genotype which has previously been associated with expression of *Oxtr*-H (Danoff et al., 2021). The number of offspring included in each group following filtering for genotype are: saline treatment: 7 males, 7 females; low dose oxytocin: 6 males, 6 females; medium dose oxytocin: 6 males, 7 females; high dose oxytocin: 5 males, 6 females; no treatment control: 7 males, 8 females. 1 μg RNA from fetal midbrain was processed for cDNA synthesis using the iScript cDNA synthesis kit (Bio-Rad) according to manufacturer instruction. Reverse transcriptase quantitative PCR (RT-qPCR) was completed using the CFX966 system (Bio-Rad) with Power SYBR Green chemistry (Applied Biosystems). Primers were designed to capture distinct transcripts or sets of transcripts. We note that we attempted to generate an assay specific to transcripts *Oxtr*-K and *Oxtr*-L, and while PCR was successful and generated the correct amplicon, the assay could not be made efficient enough for quantitation, likely because of low abundance of these transcripts in brain tissue. Expression of all transcripts were normalized to *Gapdh* using the comparative Ct method, 2^−ΔCt^. See Supplementary Table S1 for all primers and PCR conditions. Specificity of products was confirmed using melt curve analysis. All RT-qPCR reactions were completed in triplicate, and the average Ct value of replicates was used for further analysis.

### Genotyping of SNP KLW2 in induction of labor samples

5 ng of DNA was used as template for PCR using a Pyromark PCR kit (Qiagen, Valencia, CA) according to manufacturer instructions using 0.2 μM forward primer 5′-GGA​CCC​AAG​GCC​TAT​GTC​A-3′ and biotinylated reverse primer 5′-ATG​AGC​TTG​ACG​CTA​CTG​ACT​CG-3’. The following cycling conditions were used for amplification of the target fragment: [Step 1: (95°C/15 min)/1 cycle, Step 2: (94°C/30 s, 58°C/30 s, 72°C/30 s)/45 cycles, Step 3: (72°C/10 min)/1 cycle, Step 4: 4°C hold. Pyrosequencing was completed using Pyromark Gold Q24 reagents (Qiagen) according to manufacturer instructions with sequencing primer 5′-TCA​AGA​TCT​GGC​AGA​AC-3’.

### DNA methylation analysis of Oxtr in induction of labor samples

Methods for measurement of DNA methylation in induction of labor samples have previously been reported (Kenkel et al., 2019). Briefly, 200 ng genomic DNA were subject to bisulfite treatment (Kit MECOV50, Invitrogen, Carlsbad, CA) per manufacturer instructions. 12 ng of bisulfite converted DNA was used as a template for PCR using a Pyromark PCR kit (Qiagen) and 0.2 uM of primers 5′-GGG​GAT​AGG​ATG​GTT​AGT​TAG​TAT​T-3′ and 5′- CCA​ACA​ACC​TCA​AAA​CTC​TAC​T-3’. The following cycling conditions were used for amplification of the target *Oxtr* fragment, which included CpG sites −934_1, −934_2, −924, and −901: [Step 1: (95°C/15 min)/1 cycle, Step 2: (94°C/30 s, 58°C/30 s, 72°C/30 s)/50 cycles, Step 3: (72°C/10 min)/1 cycle, Step 4: 4°C hold. Pyrosequencing was performed using two primers: 5′-GAG​GGA​AGG​TTT​TGG​AGT​TTT​TTA​TAT-3′, which covers CpG sites −934_1, −934_2, and −924, and 5′-AGG​GAT​TGA​AAA​GTG​A-3′, which covers CpG site −901, on a Pyromark Q24 using PyroMark Gold Q24 Reagents (Qiagen) per the manufacturer protocol. Epigenotypes reported are an average of the three replicates.

### Statistical analysis

Statistical analysis was completed in R statistical software. RT-qPCR data was analyzed using mixed-effect linear models with fixed effects of a treatment by sex interaction and random effects of litter ID to account for genetic relatedness using the *afex* package in R (Team, 2018; Singmann et al., 2022). Post-hoc tests were completed used the *emmeans* R package and a modified Dunnett correction (“dunnetx”) for multiple comparisons (Lenth, 2022). Comparison of RT-qPCR data from the no treatment and saline groups were completed using the same model structure. Since no sex or treatment by sex effects were associated with expression of any transcript, the relationship between DNA methylation and *Oxtr* transcript expression was examined using mixed-effect linear models with fixed effects of DNA methylation at a specific CpG site and treatment and random effects of litter ID. Separate models were constructed for each CpG site measured to avoid collinearity.

## Results

### 5′ RACE in prairie vole brain and uterus reveals seven Oxtr transcripts

Using 5′ RACE, we identified seven *Oxtr* transcripts with unique 5′ transcription start sites in prairie vole nucleus accumbens and uterine tissue (Figures 1A, B). Though we are interested in transcriptional products of *Oxtr* in the brain, we also used two uterine samples, a virgin uterus and a postpartum uterus (day of birth), because of the abundance of *Oxtr* expression in this tissue. Four of these transcripts were previously identified in mouse hippocampus (Towers et al., 2018) and are named according to their homology. These include *Oxtr-A*, which is considered the canonical transcript, consisting of four exons and three introns. *Oxtr-B* consists of a 5′ extended exon 3 spliced to exon 4. *Oxtr-E* consists of a truncated exon 3 spliced to exon 4. *Oxtr-H* consists of a novel exon originating from the third intron of the gene spliced to exon 4. Three novel transcripts were also identified. *Oxtr-J* which originates in the middle of exon 3 and is spliced to exon 4. *Oxtr-K* and *Oxtr-L* both consist of two novel exons about 4,000 basepairs upstream of the canonical transcription start site, which get spliced to exon 2. *Oxtr-L* differs from *Oxtr-K* in that first exon is 35 bp shorter and the intron between exon 2 and 3 is retained, though we cannot rule out the possibility that *Oxtr-L* represents a transcript similar to *Oxtr-K* that has not been fully spliced. Sequences of each transcript are provided in Supplementary Data.

We predicted putative translation start sites using the ATG in the transcript sequence that produces the longest peptide sequence, which was always in frame. We compared this predicted sequence to the prairie vole OXTR protein sequence (Uniprot E0V872) to generate schematics of the predicted protein. The predicted protein products of these transcripts are shown in Figure 1C. *Oxtr-A*, *Oxtr-B*, *Oxtr-K*, and *Oxtr-L* would all make a full length OXTR protein if translated. The other transcripts could be translated to truncated OXTR proteins as shown in Figure 1C. Importantly, we do not yet have evidence that these transcripts are all translated; it is possible that some transcripts are not translated and possess gene regulatory functions or do not have any biological function.

### Maternal oxytocin administration at term pregnancy impacts expression of specific Oxtr transcripts in the fetal brain

**FIGURE 2:**
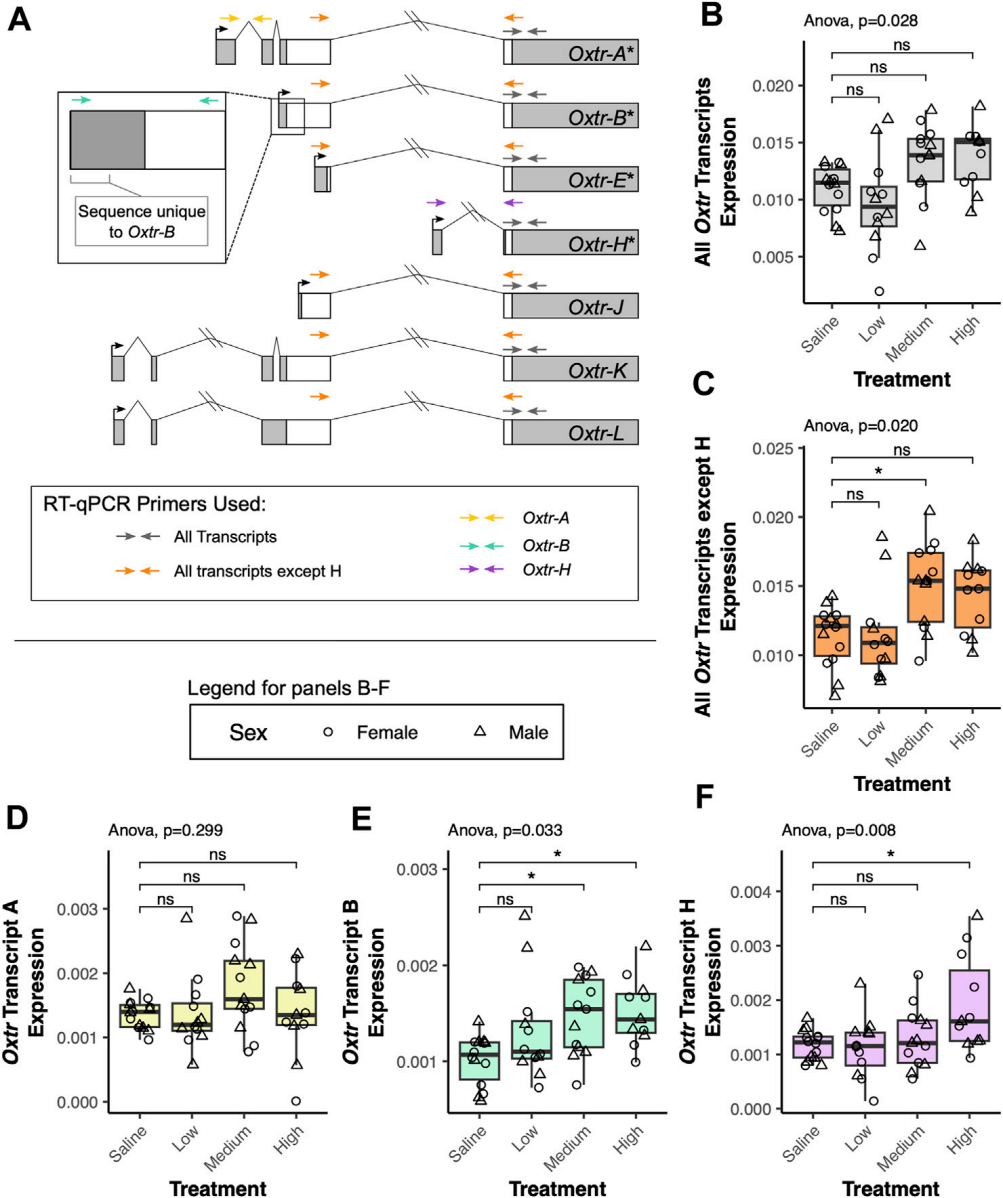
Maternal oxytocin treatment leads to increased expression of a subset of *Oxtr* transcripts in the fetal brain. **(A)** Schematic of *Oxtr* transcripts identified by 5′ RACE. Transcripts with asterisks indicate transcripts previously identified in mouse hippocampus. Primers used for RT-PCR are indicated by colored arrows with each color representing a different transcript or set of transcripts captured by the assay. **(B)** Maternal oxytocin treatment leads to differential expression of *Oxtr* when primers capture all transcripts [F_(3,22.5)_ = 3.66, *p* = 0.028]. Post-hoc tests do not indicate significant differences between any treatment group and saline after correction for multiple comparisons. **(C)** Maternal oxytocin treatment leads differential expression of *Oxtr* when measuring all transcripts except H [F_(3,22.4)_ = 4.03, *p* = 0.020]. Post-hoc tests indicate that the medium dose group differs from saline [t_(21.8)_ = 2.80, *p* = 0.029]. **(D)** Maternal oxytocin treatment does not lead to differential expression of *Oxtr* transcript A [F_(3,22.5)_ = 1.30, *p* = 0.229]. **(E)** Maternal oxytocin treatment leads differential expression of *Oxtr* transcript B [F_(3,21.7)_ = 3.50, *p* = 0.033]. Post-hoc tests indicate that the medium dose group differs from saline [t_(21.6)_ = 2.63, *p* = 0.041] and that the high dose group differs from saline [t_(21.9)_ = 2.89, *p* = 0.023]. **(F)** Maternal oxytocin treatment leads differential expression of *Oxtr* transcript H [F_(3,42)_ = 4.49, *p* = 0.008]. Post-hoc tests indicate that the high dose group differs from saline [t_(21.6)_ = 3.15, *p* = 0.013]. * *p* < 0.05, ns *p* > 0.05.

We investigated how transcriptional dynamics of *Oxtr* isoforms in the fetal brain are impacted by biological context using a model of labor induction (Kenkel et al., 2019). In this study, we injected pregnant dams with saline, low dose oxytocin (0.125 mg/kg), medium dose oxytocin (0.25 mg/kg), or high dose oxytocin (0.5 mg/kg) on the expected day of birth. An additional group of pregnant dams were not injected and used as a no treatment control. 90 min after the injection, fetuses were extracted and the fetal brain was removed. Our previous results indicate that maternal oxytocin treatment leads to increased *Oxtr* DNA methylation and impacts *Oxtr* expression (Kenkel et al., 2019). We suspected that a relationship between maternal oxytocin exposure and *Oxtr* gene expression in the fetal brain might be further clarified by examining single transcript specific expression. We investigated this using reverse transcriptase quantitative PCR (RT-qPCR) with primers that can detect distinct alternative transcripts (*Oxtr*-A, *Oxtr*-B, *Oxtr*-H) or groups of transcripts (*Oxtr-All*, *Oxtr-All* except *H*) (Figure 2A).

Our results indicate that there are significant group differences in expression when using primers that capture all *Oxtr* transcripts, but *post hoc* tests do not indicate that any specific group differs from saline [F_(3,22.5)_ = 3.66, *p* = 0.028, Figure 2B]. When using primers that capture all *Oxtr* transcripts except *Oxtr-H*, there is again a significant group difference, with the medium group having increased expression compared to saline [F_(3,22.4)_ = 4.03, *p* = 0.020, Figure 2C]. We then examined if expression of only specific *Oxtr* transcripts is altered by maternally administered oxytocin. None of the treatment group expression differences in *Oxtr* transcripts can be attributed to *Oxtr-A*, which does not change expression levels with maternal oxytocin administration [F_(3,22.5)_ = 1.30, *p* = 0.229, Figure 2D]. However, these changes appear to be driven by changes in expression of *Oxtr-B*, which does significantly differ by maternal oxytocin administration, and is elevated in the medium dose and high dose groups [F_(3,21.7)_ = 3.50, *p* = 0.033, Figure 2E]. We also identify group differences in expression of *Oxtr-H*, where expression is elevated in the high dose group [F_(3,42)_ = 4.49, *p* = 0.008, Figure 2F]. Importantly, expression of each of these transcripts or groups of transcripts does not differ between offspring of saline treated dams and offspring of dams receiving no injection (Supplementary Figure S1). These results indicate that maternal oxytocin treatment alters expression of specific *Oxtr* transcripts in the fetal brain in a dose-dependent manner and suggests the importance of clarifying expression of these specific transcripts in previous literature in the vole and the mouse. Moreover, these results underscore the importance of precise quantification of specific transcripts when examining how endogenous or exogenous factors impact *Oxtr* expression in the brain.

### DNA methylation in the MT2 region of Oxtr is negatively associated with Oxtr-B expression

**FIGURE 3:**
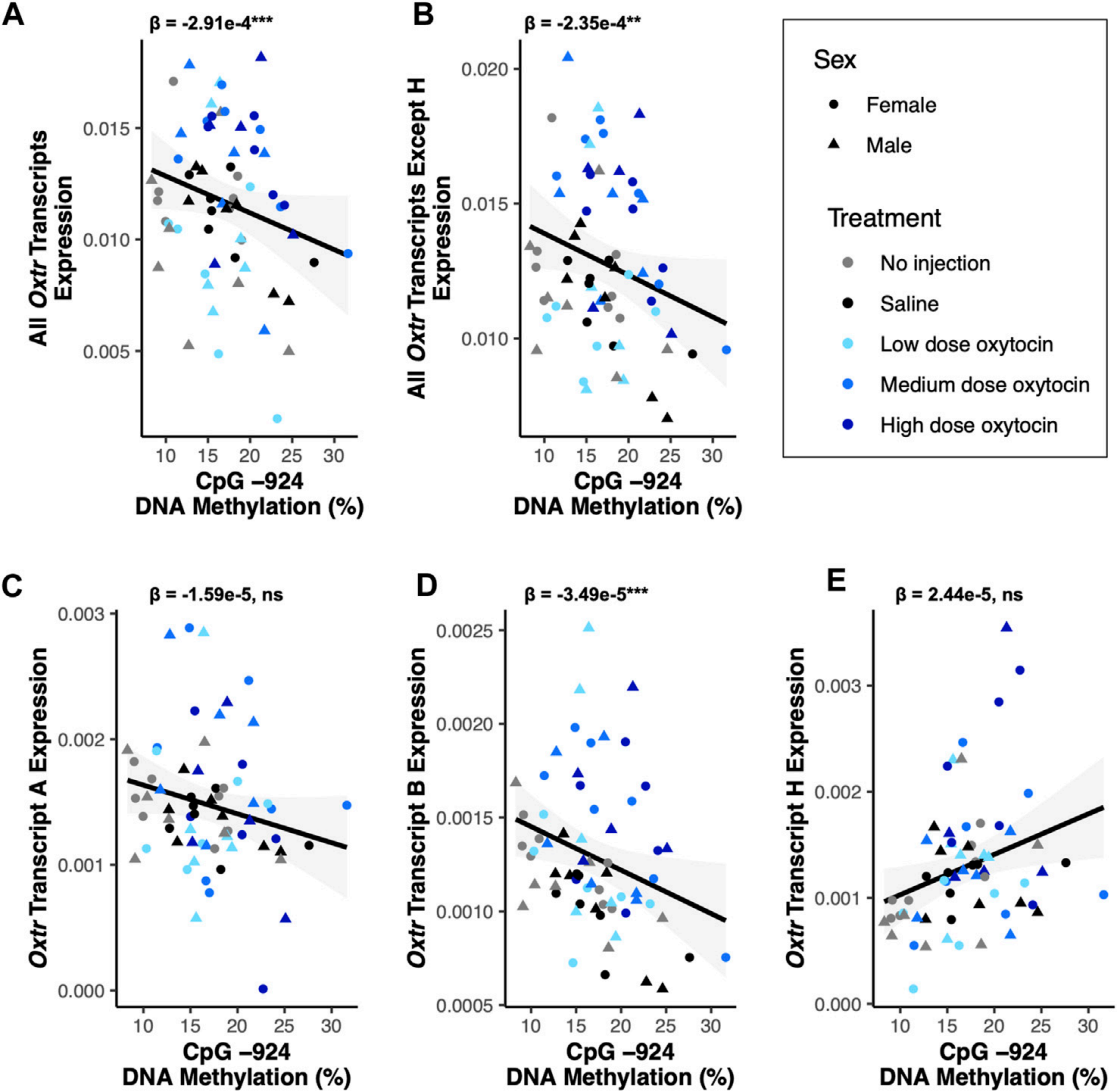
DNA methylation of MT2 in the fetal brain is associated with *Oxtr*-B expression but not *Oxtr*-A or *Oxtr*-H. For all plots, β values for CpG −924 DNA methylation from mixed model regressions which account for oxytocin treatment group and litter effects are shown. Regression lines are shown for visualization purposes. **(A)** DNA methylation at CpG −924 is negatively associated with expression of all *Oxtr* transcripts [F_(1,51.6)_ = 12.55, *p* < 0.001]. **(B)** DNA methylation at CpG −924 is negatively associated with expression of all *Oxtr* transcripts except H [F_(1,58.7)_ = 11.28, *p* = 0.001]. **(C)** DNA methylation at CpG −924 is not associated with expression of *Oxtr* transcript A [F_(1,59.0)_ = 1.29, *p* = 0.26]. **(D)** DNA methylation at CpG −924 is negatively associated with expression of *Oxtr* transcript B [F_(1,54.4)_ = 14.15, *p* < 0.001]. **(E)** DNA methylation at CpG −924 is not associated with expression of *Oxtr* transcript H [F_(1,59.0)_ = 2.44, *p* = 0.123].

DNA methylation is an epigenetic modification that occurs when a methyl group is covalently added to cytosine, typically in a cytosine-phosphate-guanine (CpG) context (Bird, 2011). DNA methylation in gene promoters is generally associated with transcriptional repression (Bird, 2011). DNA methylation in a regulatory region of *Oxtr* called MT2, which is located within exon 1 and intron 1 of *Oxtr*, is associated with reduced transcription of *OXTR* in both humans and prairie voles (Kusui et al., 2001; Danoff et al., 2021). We tested if DNA methylation in MT2 regulates specific *Oxtr* transcripts. We examined DNA methylation at four CpG sites in MT2 (CpGs −934_1, −934_2, −924, and −901) which we have previously shown to be sensitive to early life experience and are among the CpG sites where methylation is most highly associated with *Oxtr* expression (Danoff et al., 2021). Results of all DNA methylation models are in Supplementary Table S2 and indicate that all CpG sites measured show similar relationships with *Oxtr* transcript expression. For simplicity, results from CpG site −924 are provided below and in Figure 3. DNA methylation at CpG site −924 is negatively associated with expression of *Oxtr* when the assay does not distinguish between individual transcripts [F_(1,51.6)_ = 12.55, *p* < 0.001; Figure 3A] and *Oxtr* expression when the assay measures all transcripts except H [F_(1,58.7)_ = 11.28, *p* = 0.001; Figure 3B]. We next measured the expression of specific transcripts to identify which transcripts are associated with MT2 DNA methylation. In the fetal brain, the relationship between *Oxtr* gene expression and DNA methylation cannot be attributed to regulation of *Oxtr-A*, which is not associated with DNA methylation at CpG −924 [F_(1,59.0)_ = 1.29, *p* = 0.26; Figure 3C]. However, DNA methylation at CpG −924 is negatively associated with expression of *Oxtr-B* [F_(1,54.4)_ = 14.15, *p* < 0.001; Figure 2D]. DNA methylation at CpG −924 is not associated with expression of *Oxtr-H* [F_(1,59.0)_ = 2.44, *p* = 0.123; Figure 2E]. These results indicate that DNA methylation in MT2 impacts expression of *Oxtr*-B in the fetal brain.

## Discussion

We provide evidence of seven alternative transcripts of *Oxtr* in prairie voles in multiple tissues. Four of these transcripts were previously identified in mouse hippocampus, indicating conservation of these transcripts across species. An additional three transcripts were identified which are novel. We confirmed the presence of five of these transcripts with RT-qPCR in the fetal brain. Putative protein product sequence-based prediction indicates that while four of these transcripts are predicted to produce full length OXTR protein, the remaining three would make partial protein products. Possible functions of these isoforms are discussed below, though we have not provided evidence that these transcripts are translated into protein. Further investigation of these transcripts is required to examine functional roles on oxytocin-dependent processes including birth and social behavior.

Four of the described isoforms contain the entire protein coding sequence and could therefore be translated into the full length OXTR protein. It is possible that there is context- or tissue-dependent expression of particular transcripts which might relate to the regulation or function of OXTR and other receptors known to bind oxytocin. In other GPCRs, it has been shown that transcripts with extended 5′ untranslated regions are translated less efficiently and may allow for a quick context-dependent change in expression of a gene that is readily reversible (Kreth et al., 2008). This mechanism may be the functional effect of *Oxtr-K* and *Oxtr-L* expression, allowing the brain and/or uterus to slow *Oxtr* translation while keeping pools of mRNA encoding for full length Oxtr in the cell.

If the transcripts which do not include the entire protein-coding region are indeed translated, these truncated OXTR proteins may alter the availability or activity of the full-length protein or other closely related receptors known to bind to oxytocin (e.g., AVPR1A, AVPR1B). It is well documented in other GPCRs that truncated receptors regulate both the activity and availability of full-length receptors (Markovic and Challiss, 2009; Liu et al., 2021). Crystal structure data suggest an important modulatory role for the seventh transmembrane domain of OXTR, indicating that translated *Oxtr-E* and *Oxtr-H* may regulate oxytocin binding capacity (Meyerowitz et al., 2022). OXTR homodimerizes and heterodimerizes with other GPCRs, and interacts with many G proteins and β-arrestin at its C-terminus, all of which have implications for oxytocin signaling (Oakley et al., 2001; Busnelli and Chini, 2018). Truncated forms of OXTR containing the C terminus in the membrane or cytoplasm could bind these proteins and make them unavailable to full-length OXTR and other GPCRs. Altogether, the presence of multiple truncated forms of OXTR proteins would have wide implications for the function of OXTR at the cellular level.

It is also possible that many of the alternative isoforms are not translated into protein but instead act as regulatory RNAs for the *Oxtr* gene. Many protein coding genes express non-coding alternative isoforms with regulatory function (Dhamija and Menon, 2018). These functions can either increase or decrease expression of the canonical transcript or be independent of the canonical transcript’s function. Given that we have previously shown correlation between the canonical *Oxtr* transcript and *Oxtr*-H, the described isoforms might increase expression of the canonical transcript (Danoff et al., 2021). Further investigation using methods to knockdown or knockout specific *Oxtr* transcripts will provide insight as to the function of these transcripts.

Finally, using samples from a study simulating labor induction, we show that specific *Oxtr* transcripts are sensitive to maternal oxytocin administration in a dose-dependent manner. Most effects in our assays which measure expression of multiple transcripts can be attributed to increased expression of *Oxtr-B* in the medium and high dose oxytocin administration groups. While *Oxtr-B* has increased expression in both the medium and high dose groups, *Oxtr*-H only has increased expression in the high dose group, suggesting that this transcript might be invoked in situations where the brain is responding to an abundance of oxytocin signaling. Notably, previous work in mice suggested that *Oxtr-B* is the transcript most tightly regulated by DNA methylation (Towers et al., 2018). Our results provide further evidence that DNA methylation in MT2 regulates expression of *Oxtr-B* specifically. DNA methylation in MT2 at least in the fetal period is not associated with expression of transcripts *Oxtr-A* and *Oxtr-H*. Altogether, these results suggest that *Oxtr-B* may be the dominant transcript expressed in the brain, and is the most sensitive to changes in the environment and local epigenetic state.

Our study does not present an exhaustive list of *Oxtr* transcripts. The gene-specific primer we used for amplification of 5′ RACE products binds to the fourth exon of transcript *Oxtr-A*. As such, we only identified transcripts containing this exon and cannot exclude the possibility that there are transcripts which do not include this exon. Additionally, we may not have sequenced enough clones to capture very rare transcripts. Nonetheless, the results presented here provide evidence of seven alternative isoforms of *Oxtr* in prairie voles. These isoforms represent alternative *Oxtr* gene products which may modulate the activity of full length OXTR in one of three ways: by producing proteins which interact with (1) the full-length OXTR, (2) other receptors that dimerize with OXTR, or (3) other proteins that interact with OXTR such as G proteins and β-arrestin. These products might also alter the transcriptional or translational ability of the canonical transcript. This has widespread implications for understanding regulation of oxytocin signaling in many tissues. Studies measuring expression of *Oxtr* in prairie voles should consider which of these transcripts the chosen quantification method measures for proper interpretation. Interpretation of such results should also consider that context-dependent expression of these isoforms may allow the cell to quickly and reversibly modulate oxytocin signaling in response to both internal processes such as parturition or external events such as social interaction without fully adjusting the regulation of the *Oxtr* gene. Further studies are necessary to examine the functional role of each of these transcripts in the cell and how the expression of each transcript is modulated by biological and environmental context. In the context of the uterus, for example, specific transcripts might be critical for contractions during birth which require oxytocin signaling, a process which might be disrupted if gene regulation goes awry. In the brain, there might be more subtle variation in transcript use which is related to specific environmental exposures, experiences, or developmental trajectories. Future studies which examine the functional characteristic of different *Oxtr* transcripts in the brain should also study such factors, which may explain why some transcripts are upregulated and others are not. Lastly, it has not escaped our notice that critical SNP findings in the human literature identify a prominent effect of a polymorphism in the third intron of *OXTR* on many behavioral and psychiatric outcomes (Wu et al., 2005; Li et al., 2015; Gong et al., 2017). Follow up work in the human to identify the presence of alternative OXTR transcripts is needed, especially transcript H, which would have a transcription start site near commonly studied *OXTR* SNPs if present in humans.

## Data Availability

Transcript sequences are deposited in GenBank with the following accession numbers: OR044891, OR044892, OR044893, OR044894, OR044895, OR044896, OR044897. Sequences are also available in Supplementary Data. All other data is available upon request from the corresponding author.
