# Supplementary material for: Transcriptional diversity of the oxytocin receptor in prairie voles: mechanistic implications for behavioral neuroscience and maternal physiology
Source: Front Genet. 2023 Aug 29;14:1225197. doi: 10.3389/fgene.2023.1225197 (PMC10495980; doi:10.3389/fgene.2023.1225197)
Supplement: Supplementary file 1 [file DataSheet2.docx]

**Supplementary Information**

**
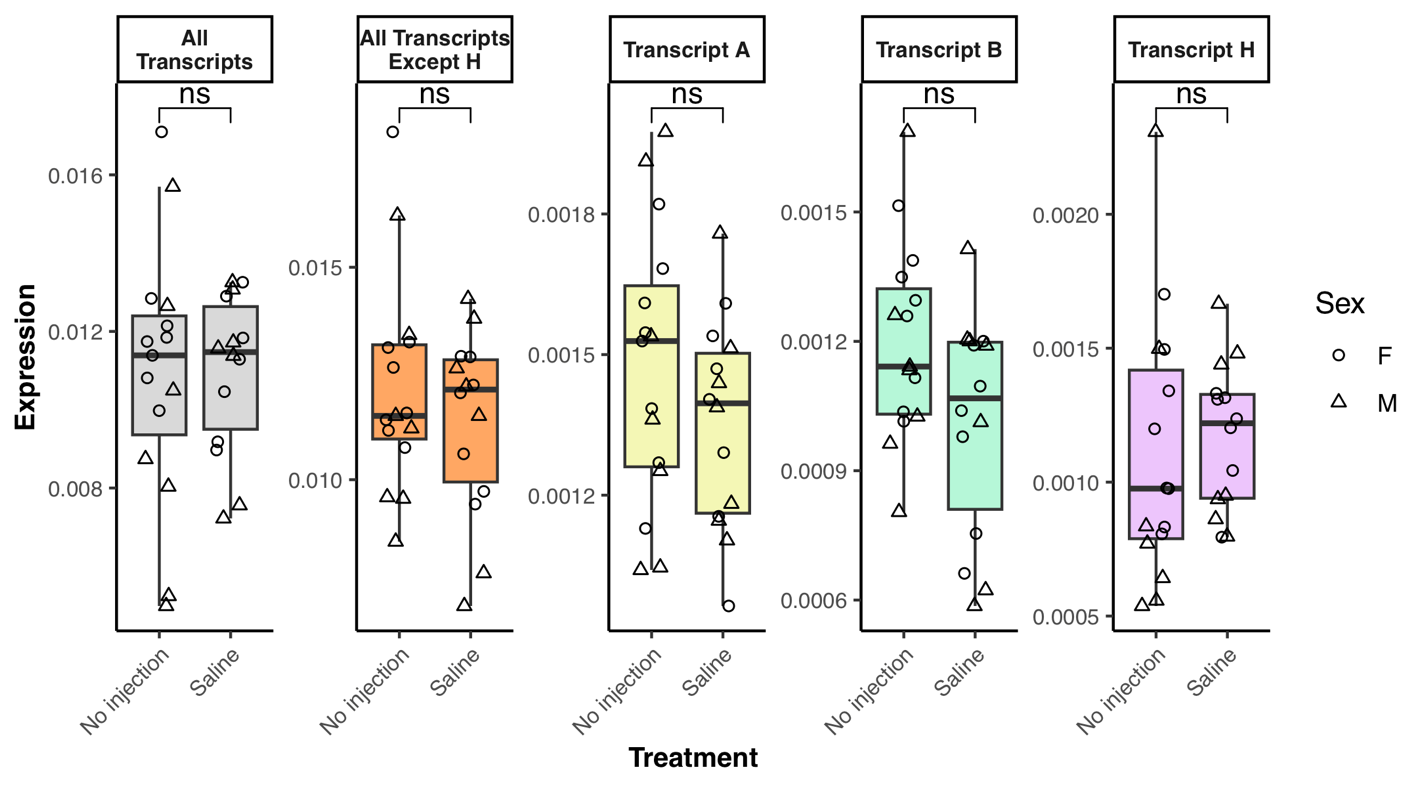
**

**Fig. S1. No difference in *Oxtr* transcript expression in offspring of saline-injected dams and untreated dams.**

Expression of all transcripts was compared between the no treatment group and saline treatment groups in the induction of labor study. There were no differences in any transcript (All *Oxtr* transcripts, F_(1,11.34)_=0.002, *p*=0.967; All *Oxtr* transcripts except H, F_(1,10.85)_=0.071, *p*=0.418; *Oxtr* transcript A, F_(1,13.02)_=1.09, *p*=0.316; *Oxtr* transcript B, F_(1,13.12)_=2.83, *p*=0.116; *Oxtr* transcript H, F_(1,13.12)_=0.21, *p*=0.651).

**Table S1. RT-qPCR Primers and Conditions**

| Transcript | Primers | PCR Conditions | | Efficiency |
| --- | --- | --- | --- | --- |
| All *Oxtr* transcripts | F: 5’- CCTTCATCATCGCCATGCTC-3’  R: 5’- AGAAAGCGCTGCACAAGTTC-3’ | 95° C, 10 min | 1 cycle | E: 99.1%  R^2^: 0.984 |
|  |  | 95° C, 15 sec | 40 cycles |  |
|  |  | 62.5° C, 1 min |  |  |
| All *Oxtr* transcripts except H | F: 5′-GCCTTTCTTCTTCGTGCAGATG-3′  R: 5′-ATGTAGATCCAGGGGTTGCAG-3′ | 95° C, 10 min | 1 cycle | E: 99.7%  R^2^: 0.993 |
|  |  | 95° C, 15 sec | 35 cycles |  |
|  |  | 63.4° C, 1 min |  |  |
| *Oxtr* transcript A | F: 5’- GGCAGCTCTCCCATCCTG-3’  R: 5’- CAAGATGCAGACCTCCTTTGA-3’ | 95° C, 10 min | 1 cycle | E: 103.7%  R^2^: 0.944 |
|  |  | 95° C, 15 sec | 40 cycles |  |
|  |  | 62.5° C, 1 min |  |  |
| *Oxtr* transcript B | F: 5’- GTTTGTTTCAGGGCGGAAGG-3’  R: 5’- TCCAACTCGAAGCTCCAGTT-3’ | 95° C, 10 min | 1 cycle | E: 99.1%  R^2^: 0.984 |
|  |  | 95° C, 15 sec | 40 cycles |  |
|  |  | 62.5° C, 1 min |  |  |
| *Oxtr* transcript H | F: 5’- TTTTTGCTTTCCCAATGTGC-3’  R: 5’- CCGTGAACAGCATGTAGATCCA-3’ | 95° C, 10 min | 1 cycle | E: 100.3%  R^2^: 0.971 |
|  |  | 95° C, 15 sec | 40 cycles |  |
|  |  | 59.5° C, 1 min |  |  |
| *Gapdh* | F: 5’- TTGCCCGTTGACTTTGTCAC-3’  R: 5’- GCCACAGCCTCAGCATATTTC-3’ | 95° C, 10 min | 1 cycle | E: 93.5%  R^2^: 0.998 |
|  |  | 95° C, 15 sec | 35 cycles |  |
|  |  | 63.4° C, 1 min |  |  |

**Table S2. Results from models examining DNA methylation in MT2 and oxytocin treatment in regulation of specific *Oxtr* transcripts.**

| **Model formula: OxtrAll ~ CpG_934_1 + Treatment + (1\|Litter)** | | | |
| --- | --- | --- | --- |
| Variable | F statistic | DF | p value |
| CpG_934_1 | 9.45 | (1, 51.1) | 0.003 |
| Treatment | 5.44 | (4, 27.6) | 0.002 |
| **Model formula: OxtrAll ~ CpG_934_2 + Treatment + (1\|Litter)** | | | |
| Variable | F statistic | DF | p value |
| CpG_934_2 | 3.06 | (1, 53.8) | 0.086 |
| Treatment | 4.27 | (4, 26.6) | 0.009 |
| **Model formula: OxtrA ll~ CpG_924 + Treatment + (1\|Litter)** | | | |
| Variable | F statistic | DF | p value |
| CpG_924 | 12.55 | (1, 51.6) | <0.001 |
| Treatment | 6.16 | (4, 27.3) | 0.001 |
| **Model formula: OxtrAll ~ CpG_901 + Treatment + (1\|Litter)** | | | |
| Variable | F statistic | DF | p value |
| CpG_901 | 9.92 | (1, 56.3) | 0.003 |
| Treatment | 4.99 | (4, 27.9) | 0.004 |
| **Model formula: OxtrAllButH ~ CpG_934_1 + Treatment + (1\|Litter)** | | | |
| Variable | F statistic | DF | p value |
| CpG_934_1 | 8.83 | (1, 58.9) | 0.004 |
| Treatment | 5.22 | (4, 28.1) | 0.003 |
| **Model formula: OxtrAllButH ~ CpG_934_2 + Treatment + (1\|Litter)** | | | |
| Variable | F statistic | DF | p value |
| CpG_934_2 | 3.10 | (1, 59.0) | 0.084 |
| Treatment | 4.54 | (4, 26.3) | 0.006 |
| **Model formula: OxtrAllButH ~ CpG_924 + Treatment + (1\|Litter)** | | | |
| Variable | F statistic | DF | p value |
| CpG_924 | 11.28 | (1, 58.7) | 0.001 |
| Treatment | 5.88 | (4, 27.2) | 0.002 |
| **Model formula: OxtrAllButH ~ CpG_901 + Treatment + (1\|Litter)** | | | |
| Variable | F statistic | DF | p value |
| CpG_901 | 9.20 | (1, 58.6) | 0.004 |
| Treatment | 4.89 | (4, 27.7) | 0.004 |
| **Model formula: OxtrA ~ CpG_934_1 + Treatment + (1\|Litter)** | | | |
| Variable | F statistic | DF | p value |
| CpG_934_1 | 0.58 | (1, 59.0) | 0.448 |
| Treatment | 1.23 | (4, 28.7) | 0.322 |
| **Model formula: OxtrA ~ CpG_934_2 + Treatment + (1\|Litter)** | | | |
| Variable | F statistic | DF | p value |
| CpG_934_2 | 0.62 | (1, 58.9) | 0.434 |
| Treatment | 1.26 | (4, 28.0) | 0.308 |
| **Model formula: OxtrA ~ CpG_924 + Treatment + (1\|Litter)** | | | |
| Variable | F statistic | DF | p value |
| CpG_924 | 1.29 | (1, 59.0) | 0.260 |
| Treatment | 1.32 | (4, 28.0) | 0.288 |
| **Model formula: OxtrA ~ CpG_901 + Treatment + (1\|Litter)** | | | |
| Variable | F statistic | DF | p value |
| CpG_901 | 0.64 | (1, 58.4) | 0.428 |
| Treatment | 1.19 | (4, 28.3) | 0.337 |
| **Model formula: OxtrB ~ CpG_934_1 + Treatment + (1\|Litter)** | | | |
| Variable | F statistic | DF | p value |
| CpG_934_1 | 8.98 | (1, 55.4) | 0.004 |
| Treatment | 5.24 | (4, 29.3) | 0.003 |
| **Model formula: OxtrB ~ CpG_934_2 + Treatment + (1\|Litter)** | | | |
| Variable | F statistic | DF | p value |
| CpG_934_2 | 5.79 | (1, 54.7) | 0.019 |
| Treatment | 5.34 | (4, 27.9) | 0.003 |
| **Model formula: OxtrB ~ CpG_924 + Treatment + (1\|Litter)** | | | |
| Variable | F statistic | DF | p value |
| CpG_924 | 14.15 | (1, 54.4) | <0.001 |
| Treatment | 6.26 | (4, 29.0) | <0.001 |
| **Model formula: OxtrB ~ CpG_901 + Treatment + (1\|Litter)** | | | |
| Variable | F statistic | DF | p value |
| CpG_901 | 13.75 | (1, 56.7) | <0.001 |
| Treatment | 5.68 | (4, 29.1) | 0.002 |
| **Model formula: OxtrH ~ CpG_934_1 + Treatment + (1\|Litter)** | | | |
| Variable | F statistic | DF | p value |
| CpG_934_1 | 0.96 | (1, 59.0) | 0.332 |
| Treatment | 3.33 | (4, 59.0) | 0.016 |
| **Model formula: OxtrH ~ CpG_934_2 + Treatment + (1\|Litter)** | | | |
| Variable | F statistic | DF | p value |
| CpG_934_2 | 8.23 | (1, 59.0) | 0.006 |
| Treatment | 1.94 | (4, 59.0) | 0.116 |
| **Model formula: OxtrH ~ CpG_924 + Treatment + (1\|Litter)** | | | |
| Variable | F statistic | DF | p value |
| CpG_924 | 2.44 | (1, 59.0) | 0.123 |
| Treatment | 3.30 | (4, 59.0) | 0.017 |
| **Model formula: OxtrH ~ CpG_901 + Treatment + (1\|Litter)** | | | |
| Variable | F statistic | DF | p value |
| CpG_901 | 1.25 | (1, 59.0) | 0.268 |
| Treatment | 3.52 | (4, 59.0) | 0.012 |
