# Supplementary material for: Transcriptional diversity of the oxytocin receptor in prairie voles: mechanistic implications for behavioral neuroscience and maternal physiology
Source: Front Genet. 2023 Aug 29;14:1225197. doi: 10.3389/fgene.2023.1225197 (PMC10495980; doi:10.3389/fgene.2023.1225197)
Supplement: Supplementary file 2 [file DataSheet1.docx]

Supplementary Data. Alternative 5’ Transcription Start Sites (TSS) for each *Oxtr* variant

*note: if multiple start sites were identified for each variant, the longest transcript is listed and alternative 5’ starts are underlined; All 3’ ends are anchored by an Oxtr specific primer sequence, if sequencing reached this primer it is indicated bold; putative polymorphisms are identified in red; predicted translation start is indicated in green; confirmed splice site boundary (/), potential splice site boundary (/). If sequence is in italics, it was not confirmed by sequencing (see K and L). An intron that is maintained in transcript L is indicated in blue.

**>*Oxtr-A***

5’−GTGCTGACGCTGTCCGTGCACAGCCTGGCCACCGCTCTTCCCTTGCCTTCGGTCCGCATCCAGCCACCAGCAAAGCCACAGACTAGAGGACTGCAAAGACGCTGGGATCTACTCGTGGTGGCACCGAGCGTGGCGCCGGCAGCTCTCCCATCCTGGGCGTAGAGCCCGCGCATCTCTGAGGCCATCCGCTGCGGTGCAACTTCCCCAGTGGCGAGGCAACTTTGGGCTAACCTGCAGGCTCTTTCCAG/GTAGAGCGCCTGAGAGCGCCTGACCCTTTCCC**A**GGACCCACGCTCAAGCGCATCTGCAGAGGCTCAAAGGAGGTC**CT**CATCTTGCAGAGACTCAAAGGAGATCAGGCAAACCGGGATTTCTGCGGTGGTGGCTGAGCAGCCCAGTGGCTGGGCTTGTGTCCTGAGACCCAG/GGCGGAAGGAGGCAGATCAGTGCCCGGGTGCCCCGGCAGACACCTGGACAACTCATCGGGCCCCGCCCCACGAGCCA**A**GCTTTAAAGAGCAGCAAGGCCGGGTGCTCCCTCGCGGTCGCGGTCACGGTC**ATG**GAGGGCACCCCTGCAGCCAACTGGAGCTTCGAGTTGGACCTCGGGAGTGGAGTGTCGCCGGGGGTGGAGGGCAACCTCACAGCCGGGCCACCGCAGCGCAACGAGGCCCTGGCACGCGTGGAGGTGGCGGTGCTGTGCCTCATTCTGTTCCTGGCGCTGAGCGGCAACGCGTGCGTGCTGCTGGCGCTGCGCATCACACGCCACAAGCACTCGCGCCTCTTCTTTTTCATGAAGCACCTGAGCATCGCTGACCTGGTGGTGGCTGTGTTCCAGGTGCTCCCGCAGCTGCTGTGGGACATCACCTTCCGCTTCTACGGGCCCGACCTG−3’

**>*Oxtr-B***

5’−AAGAGAAGCACAAAGATCTTCTCATTGCCCTCTCGGTTTGTTTCAGGGCGGAAGGAGGCAGATCAGTGCCCGGGTGCCCCGGCAGACACCTGGACAACTCATCGGGCCCCGCCCCACGAGCCA**A**GCTTTAAAGAGCAGCAAGGCCGGGTGCTCCCTCGCGGTCGCGGTCACGGTC**ATG**GAGGGCACCCCTGCAGCCAACTGGAGCTTCGAGTTGGACCTCGGGAGTGGAGTGTCGCCGGGGGTGGAGGGCAACCTCACAGCCGGGCCACCGCAGCGCAACGAGGCCCTGGCACGCGTGGAGGTGGCGGTGCTGTGCCTCATTCTGTTCCTGGCGCTGAGCGGCAACGCGTGCGTGCTGCTGGCGCTGCGCATCACACGCCACAAGCACTCGCGCCTCTTCTTTTTCATGAAGCACCTGAGCATCGCTGACCTGGTGGTGGCTGTGTTCCAGGTGCTCCCGCAGCTGCTGTGGGACATCACCTTCCGCTTCTACGGGCCCGACCTGCTGTGTCGTCTGGTCAAGTACTTGCA**G**GTGGTGGGCATGTTCGCTTCCACCTACCTGCTGCTGCTTATGTCGCTCGACCGCTGCCTGGCCATCTGCCAGCCGCTGCGCTCTCTGCGACGCCGAACCGACCGCCTGGCGGTGCTAGCGACATGGCTGGGCTGCCTGGTGGCCAGCGCGCCGCAGGTGCACATTTTCTCACTGCGCGAAGTGGC**A**GACGGTGTTTTTGACTGCTGGGCTGTCTTCATCCAGCCTTGGGGACCCAAGGCCTATGTCACGTGGATCACGCTTGCCGTCTACATTGTGCCTGTCATAGTGCTGGCCGCCTGCTATGGCCTCATCAGCTTCAAGATCTGGCAGAACCTGCGACTCAAGACGGCAGCGGCGGCGGCCGAGGGACTGANGNATCTGCTGCCGGTGGAGCTGGGCGTGCGGC−3’

**>*Oxtr-E***

5’−TTTTCTCACTGCGCGAAGTGGC**A**GACGGTGTTTTTGACTGCTGGGCTGTCTTCATCCAGCCTTGGGGACCCAAGGCCTATGTCACGTGGATCACGCTTGCCGTCTACATTGTGCCTGTCATAGTGCTGGCCGCCTGCTATGGCCTCATCAGCTTCAAGATCTGGCAGAACCTGCGACTCAAGACGGCAGCGGCGGCGGCCGAGGGGACTGAGGGATCTGCTGCCGGTGGAGCTGGGCGTGCGGCGCTGGCTCGAGTCAGTAGCGTCAAGCTCATCTCCAAGGCCAAGATCCGCACAGTGAAG**ATG**ACCTTCATCATTGTACTGGCCTTCATCGTGTGCTGGACGCCTTTCTTCTTCGTGCAGATGTGGAGCGTCTGGGACGTCAATGCGCCCAAGGAAG/CTTCTGCCTTCATCAT**T**GCCATGCTCTTGGCCAGCCTCAACAGCTGCTGCAACCCCTGGATCTACATGCTGTTCACGGGCCACCTCTTTCACGAACTTGTGCAGCGCTTTCTCTG**T**TGCTCTGCCCGCT**ACCTGAAGGGCAGCAGGCCCGG**−3’

**>*Oxtr-H***

5’−GAACAATCTCTCTCTCCCCACTCACC**C**CACTATCCGAGTGTGAGCTTAATTTTCCCAGAGGGAACTGCATCTGAGTCCAGTCA**T**CCTGCGAGCAGGCCATGTCCTGTCATCTAACAGGGAGATTGTCTTTTTGCTTTCCCAATGTGCTAGAG**G**AGGCGGCCGGTTTATCAAGACTTCCTGTCTGCTAATTTCATGCTTCAATTCAG/CTTCTGCCTTCATCATCGCC**ATG**CTCTTGGCCAGCCTCAACAGCTGCTGCAACCCCTGGATCTACATGCTGTTCACGGGCCACCTCTTTCACGAACTTGTGCAGCGCTTTCTCTGCTGCTCTGCCCGCT**ACCTGAAGGGCAGCAGGCCCGG**−3’

**>*Oxtr-J***

5’−ATTCTGTTCCTGGCGCTGAGCGGCAACGCGTGCGTGCTGCTGGCGCTGCGCATCACACGCCACAAGCACTCGCGCCTCTTCTTTTTC**ATG**AAGCACCTGAGCATCGCTGACCTGGTGGTGGCTGTGTTCCAGGTGCTCCCGCAGCTGCTGTGGGACATCACCTTCCGCTTCTACGGGCCCGACCTGCTGTGTCGTCTGGTCAAGTACTTGCA**G**GTGGTGGGCATGTTCGCTTCCACCTACCTGCTGCTGCTTATGTCGCTCGACCGCTGCCTGGCCATCTGCCAGCCGCTGCGCTCTCTGCGACGCCGAACCGACCGCCTGGCGGTGCTAGCGACATGGCTGGGCTGCCTGGTGGCCAGCGCGCCGCAGGTGCACATTTTCTCACTGCGCGAAGTGGC**A**GACGGTGTTTTTGACTGCTGGGCTGTCTTCATCCAGCCTTGGGGACCCAAGGCCTATGTCACGTGGATCACGCTTGCCGTCTACATTGTGCCTGTCATAGTGCTGGCCGCCTGCTATGGCCTCATCAGCTTCAAGATCTGGCAGAACCTGCGACTCAAGACGGCAGCGGCGGCGGCCGAGGGGACTGAGGGATCTGCTGCCGGTGGAGCTGGGCGTGCGGCGCTGGCTCGAGTCAGTAGCGTCAAGCTCATCTCCAAGGCCAAGATCCGCACAGTGAAGATGACCTTCATCATTGTACTGGCCTTCATCGTGTGCTGGACGCCTTTCTTCTTCGTGCAGATGTGGAGCGTCTGGGACGTCAATGCGCCCAAGGAAG/CTTCTGCCTTCATCAT**T**GCCATGCTCTTGGCCAGCCTCAACAGCTGCTGCAACCCCTGGNTCTACATGCTGTTCACGGGNCACCTCTTTCACGAACTTGTGCAGCGCTTTCTCTGTTGCTCTGCCCGCT**ACCTGAAGGGCAGCAGGCCCGG**−3’

**>*Oxtr-K***

5’−ACTCCCTGAACTTCCCAGGCCAGGCCAGTCCACATGTCCTGCTGTCCCTGGCCTCTTGCTGGCAGCAGCAAATGAAGAAGGCCAAGGGCAAGGCTGTCAGCAGCAGGAAGATTATGGCAGTGTGTTCACCACCATCAGGCCTGAGCGAGTTACTGACACCTTCTGGAGGCTGGAGCCCAAGCAGAGATGATTTCCGGGAGGGAAGAAACTGGAGGCAGTGGCCATTCATTTCCTCACTACTGCAGAGAGGCTAAATGTTCCAGAACTTGAAAACTACCAAGCTACTAAACTCCTTCCCAG/GGTTTCACTATATTGCTCTGGCTGTCCTGGAACTCATTATGTTGACTAGGCTGACCTTGGACTCACAAAGAGCTACCTGCTTATGCCTCCCAAGTGCTGGAATTAAAGATGTGGGCCACAAA/GTAGAGCGCCTGAGAGCGCCTGACCCTTTCCC**A**GG**A**CCCACGCTCAAGCGCATCTGCAGAGGCTCAAAGGAGGTC**CT**CATCTTGCAGAGACTCAAAGGAGATCAGGCAAACCGGGATTTCTGCGGTGGTGGCTGAGCAGCCCAGTGGCTGGGCTTGTGTCCTGAGACCCAG/GGCGGAAGGAGGCAGATCAGTGCCCGGGTGCCCCGGCAGACACCTGGACAACTCATCGGGCCCCGCCCCACGAGCCACGCTTTAAAGAGCAGCAAGGCCGGGTGCTCCCTCGCGGTCGCGGTCACGGTC**ATG**GAGGGCACCCCTGCAGCCAACTGGAGCTTCGAGTTGGACCTCGGGAGTGGAGTGTCGCCGGGGGTGGAGGGCAACCTCACAGCCGGGCCACCGCAGCGCAACGAGGCCCTGGCACGCGTGGAGGTGGCGGTGCTGTGCCTCATTCTGTTCCTGGCGCTGANCGGCAACGCGTGCGTGCTGCTGGCGCTGCGCATCACACGCCACAAGCACTCNCGCCTC*TTCTTTTTCATGAAGCACCTGAGCATCGCTGACCTGGTGGTGGCTGTGTTCCAGGTGCTCCCGCAGCTGCTGTGGGACATCACCTTCCGCTTCTACGGGCCCGACCTGCTGTGTCGTCTGGTCAAGTACTTGCAAGTGGTGGGCATGTTCGCTTCCACCTACCTGCTGCTGCTTATGTCGCTCGACCGCTGCC*TGGCCNTCTGCCAGCCGCTGCGCTCTCTGCGACGCCGAACCGACCGCCTGGCGGTGCTAGCGACATGGCTGGGCTGCCTGGTGGCCAGCGCGCCGCAGGTGCACATTTTCTCACTGCGCGAAGTGGCAGACGGTGTTTTTGACTGCTGGGCTGTCTTCATCCAGCCTTGGGGACCCAAGGCCTATGTCACGTGGATCACGCTTGCCGTCTACATTGTGCCTGTCATAGTGCTGGCCGCCTGCTATGGCCTCATCAGCTTCAAGATCTGGCAGAACCTGCGACTCAAGACGGCAGCGGCGGCGGCCGAGGGGACTGAGGGATCTGCTGCCGGTGGAGCTGGGCGTGCGGCGCTGGCTCGAGTCAGTAGCGTCAAGCTCATCTCCAAGGCCAAGATCCGCACAGTGAAGATGACCTTCATCATTGTACTGGCCTTCATCGTGTGCTGGACGCCTTTCTTCTTCGTGCAGATGTGGAGCGTCTGGGACGTCAATGCGCCCAAGGAAG/CTTCTGCCTTCATCATCGCCATGCTCTTGGCCAGCCTCAACAGCTGCTGCAACCCCTGGATCTACATGCTGTTCACGGGCCACCTCTTTCACGAACTTGTGCAGCGCTTTCTCTGCTGCTCTGCCCGCT**ACCTGAAGGGCAGCAGGCCCGG**−3’

**>*Oxtr-L***

5’−GTCCTGCTGTCCCTGGCCTCTTGCTGGCAGCAGCAAATGAAGAAGGCCAAGGGCAAGGCTGTCAGCAGCAGGAAGATTATGGCAGTGTGTTCACCACCATCAGGCCTGAGCGAGTTACTGACACCTTCTGGAGGCTGGAGCCCAAGCAGAGATGATTTCCGGGAGGGAAGAAACTGGAGGCAGTGGCCATTCATTTCCTCACTACTGCAGAGAGGCTAAATGTTCCAGAACTTGAAAACTACCAAGCTACTAAACTCCTTCCCAG/GGTTTCACTATATTGCTCTGGCTGTCCTGGAACTCATTATGTTGACTAGGCTGACCTTGGACTCACAAAGAGCTACCTGCTTATGCCTCCCAAGTGCTGGAATTAAAGATGTGGGCCACAAA/GTAGAGCGCCTGAGAGCGCCTGACCCTTTCCCAGGACCCACGCTCAAGCGCATCTGCAGAGGCTCAAAGGAGGTC**CT**CATCTTGCAGAGACTCAAAGGAGATCAGGCAAACCGGGATTTCTGCGGTGGTGGCTGAGCAGCCCAGTGGCTGGGCTTGTGTCCTGAGACCCAGGTAACTTCAGATTTCCAGGAAGGGGCCAGGCACGCCCCACACGCGTCCTAAGAGAAGCACAAAGATCTTCTCATTGCCCTCTCGGTTTGTTTCAGGGCGGAAGGAGGCAGATCAGTGCCCGGGTGCCCCGGCAGACACCTGGACAACTCATCGGGCCCCGCCCCACGAGCCA**A**GCTTTAAAGAGCAGCAAGGCCGGGTGCTCCCTCGCGGTCGCGGTCACGGTC**ATG**GAGGGCACCCCTGCAGCCAACTGGAGCTTCGAGTTGGACCTCGGGAGTGGAGTGTCGCCGGGGGTGGAGGGCAACCTCACAGCCGGGCCACCGCAGCGCAACGAGGCCCTGGCACGCGTGGAGGTGGCGGTGCTGTGCCTCATTCTGTTCCTGGCGCTGANCGGCAACGCGTGCGTGCTGCTGGCGCTGCGCATCACACGCCACAAGCACTCNCGCCTC*TTCTTTTTCATGAAGCACCTGAGCATCGCTGACCTGGTGGTGGCTGTGTTCCAGGTGCTCCCGCAGCTGCTGTGGGACATCACCTTCCGCTTCTACGGGCCCGACCTGCTGTGTCGTCTGGTCAAGTACTTGCAAGTGGTGGGCATGTTCGCTTCCACCTACCTGCTGCTGCTTATGTCGCTCGACCGCTGCC*TGGCCNTCTGCCAGCCGCTGCGCTCTCTGCGACGCCGAACCGACCGCCTGGCGGTGCTAGCGACATGGCTGGGCTGCCTGGTGGCCAGCGCGCCGCAGGTGCACATTTTCTCACTGCGCGAAGTGGCAGACGGTGTTTTTGACTGCTGGGCTGTCTTCATCCAGCCTTGGGGACCCAAGGCCTATGTCACGTGGATCACGCTTGCCGTCTACATTGTGCCTGTCATAGTGCTGGCCGCCTGCTATGGCCTCATCAGCTTCAAGATCTGGCAGAACCTGCGACTCAAGACGGCAGCGGCGGCGGCCGAGGGGACTGAGGGATCTGCTGCCGGTGGAGCTGGGCGTGCGGCGCTGGCTCGAGTCAGTAGCGTCAAGCTCATCTCCAAGGCCAAGATCCGCACAGTGAAGATGACCTTCATCATTGTACTGGCCTTCATCGTGTGCTGGACGCCTTTCTTCTTCGTGCAGATGTGGAGCGTCTGGGACGTCAATGCGCCCAAGGAAG/CTTCTGCCTTCATCATCGCCATGCTCTTGGCCAGCCTCAACAGCTGCTGCAACCCCTGGATCTACATGCTGTTCACGGGCCACCTCTTTCACGAACTTGTGCAGCGCTTTCTCTGCTGCTCTGCCCGCT**ACCTGAAGGGCAGCAGGCCCGG**−3’
